# BIOLOGICAL SCIENCES GSA & AGE JOINT SYMPOSIUM

**DOI:** 10.1093/geroni/igae098.1718

**Published:** 2024-12-31

**Authors:** George Sutphin, Nathan LeBrasseur

**Affiliations:** Chair; Co-Chair

## Abstract

For a fifth consecutive year, the Biological Sciences section of GSA and the leadership of the American Aging Association have partnered to identify abstracts that reflect on their shared effort to advance the understanding of mechanisms of aging and their impact on aging and disease. This symposium will discuss biomarkers and mediators of functional decline, sarcopenia, and frailty.

